# Selective Injection System into Hippocampus CA1 via Monitored Theta Oscillation

**DOI:** 10.1371/journal.pone.0083129

**Published:** 2013-12-16

**Authors:** Jyoji Tsutajima, Takato Kunitake, Yoshihiko Wakazono, Kogo Takamiya

**Affiliations:** Department of Neuroscience, University of Miyazaki, Faculty of Medicine, Miyazaki, Japan; Tokai University, Japan

## Abstract

Methods of cell biology and electrophysiology using dissociated primary cultured neurons allow *in vitro* study of molecular functions; however, analysis of intact neuronal circuitry is often preferable. To investigate exogenous genes, viral vectors are most commonly injected using a pipette that is inserted from the top of the cortex. Although there are few reports that describe the success rate of injection in detail, it is sometimes difficult to locate the pipette tip accurately within the CA1 pyramidal cell layer because the pyramidal layer is only 0.1 mm thick. In the present study, we have developed a system to inject viral vectors accurately into the mouse hippocampal CA1 pyramidal cell layer using a stereotaxic injection system with simultaneous electrophysiological monitoring of theta oscillation. The pipette tip was positioned reliably based on integrated values of the theta oscillation in the hippocampal CA1 pyramidal cell layer. This approach allows accurate injection of solutions and provides an efficient method of gene transfer using viral vectors into the hippocampus, which can be a useful tool for studies involving the molecular mechanisms of neuronal functions.

## Introduction

The hippocampus has been intensively studied as one of the most sensitive regions in terms of brain ischemia, intractable epilepsy, and Alzheimer’s disease [1-3]. It is known to play important roles in synaptic plasticity that underlies learning and memory. In particular, the CA1 region of the hippocampus has been an area of focus because of its simple neural network anatomy. For instance, a great deal of effort has been devoted to clarify the molecular mechanisms of synaptic plasticity using an *in vitro* synaptic plasticity paradigm, long-term potentiation (LTP) [4-7]. Hippocampal CA1 LTP has usually been analyzed using acute brain slices prepared from rodents; however, it is difficult to mimic LTP in dissociated cultured neurons *in vitro*, which suggests that the hippocampal LTP is expressed by complex mechanisms including glutamate receptor trafficking and network connectivity. This complex nature of LTP poses a problem for researchers who attempt to conduct a detailed study on the molecular mechanisms of synaptic plasticity [8,9]. 

*In vitro* experimental systems, such as cultured brain slices and dissociated primary neuronal cultures, provide a high degree of molecular, cellular, and electrophysiological information in basic neuroscience research. Organotypic hippocampal slice culture has been developed to study cellular functions and morphologies under normal neuroanatomical network. However, changes in the excitability of neurons during *in vitro* culture inhibit electrophysiological analysis and may cause artificial modification of structure and function [10]. Hence, direct *in vivo* experiments provide more reliable information about many neuronal functions. 

Recent advances in molecular techniques, such as gene transfer and mutant mice approaches, have provided new findings from a single cell to the whole animal level [11,12]. In addition, recent technical advance in optogenetics has accelerated our understanding of neuronal networks *in vivo*. Within this approach, it would be helpful to develop a reliable technique to introduce exogenous genes into target brain regions. However, the accurate introduction of biological materials into particular regions *in vivo* using techniques, such as plasmid gene expression vectors, viral vectors, peptides, and chemical compounds, is still unstable. 

Theta oscillations, 4-8 Hz fluctuations in the neural field potential, have been observed in various behavioral states, such as during sleep and locomotion [13]. In particular, the theta oscillations play an important role in the hippocampal network involved in memory formation [14,15], and they have also been investigated in other brain areas, such as the cortex and amygdala [16]. The amplitude and phase of the theta oscillations are synchronized in the same layer of the hippocampus and are largest in the lacunosum-moleculare region of the CA1 layer of the hippocampus. These oscillations are controlled by cholinergic neurons in the medial septum [17]. 

In many studies, researchers optimize coordinates using test injections into mouse and rat brains head-fixed in a stereotaxic frame. As long as all animals are of the same size, this method works without significant difficulties after several attempts to find suitable target coordinates. However, when animals of different sizes are used, the coordinates will vary between individual animals. In addition, brain swelling, decompression, and miniscule cortical damage during craniotomy may change the distance to the target region.

Here, we have established a stereotaxic injection system that accurately injects small amounts of liquid into the hippocampal CA1 region, utilizing simultaneous theta oscillation monitoring during insertion of the glass injection electrodes. This method provides a useful approach to introduce not only chemicals in small quantities, but also viral vector solutions to introduce exogenous genes *in vivo*. 

## Materials and Methods

### Construction of microinjection electrode and circuit

**Figure 1:**
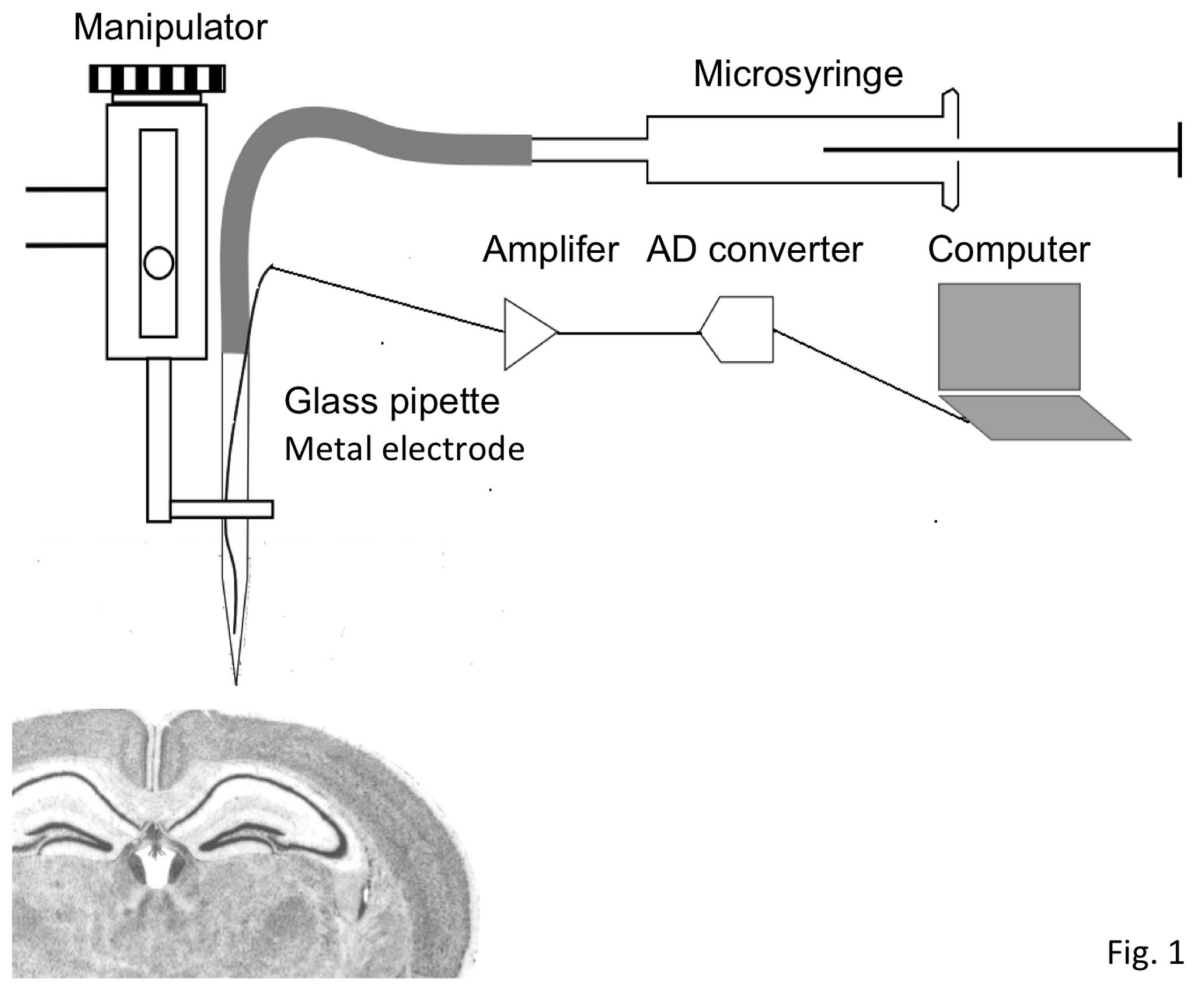
Schematic representation of the injection/recording system presented in the study. A glass pipette containing copper or Ag/AgCl EEG electrode is attached to a micro-syringe via a connection tube and is controlled by a manipulator. The electrode is connected to a personal computer to analyze the recorded signals and to calculate the real-time integrated value of the theta oscillation.

A glass pipette (1 mm outer diameter, A-M System, WA USA) was pulled to a diameter of 25 μm with an automatic puller (Sutter Instruments, SA USA) in a multi-step pulling program. The inside of the pipette was filled with either a dye or a virus solution, and the microinjection electrode was made by inserting a copper or Ag/AgCl wire into the tapered glass pipette. After confirming that the results are similar to those obtained with Ag/AgCl wire, we preferentially used a copper electrode for economical reasons. A polyethylene tube (Hibiki, Japan) was connected to the glass pipette, and the joint was melted and sealed with an open flame. The wires were pulled out of the junctions of these tubes and connected to a personal computer for electroencephalogram (EEG) analysis (Figure 1). The extended polyethylene tube and microsyringe (HAMILTON, NV USA, 10 μl) were fused, and the inside was filled with liquid paraffin without air bubbles. The microsyringe was set into a microsyringe pump (LMS, Japan) for programmed injections. Each joint part was partially melted and pasted firmly to avoid leakage. 

### Brain surgery

Four- to six-week-old C57/BL6J mice were used in the experiments. Adult male GluA1 knockout mice were used for GluA1-expressing virus injections [18]. Before the administration of anesthesia, the animals were given an intraperitoneal injection of 20 % mannitol (0.03 ml/BW) for brain decompression. In addition, to increase infection efficiency, we slowly injected the virus solution using an automatic microsyringe pump as previously reported [19,20]. Thirty minutes later, a Ketamine/Xylazine solution (2 ml of 50 mg/ml Ketamine, 0.01 g Xylazine in 10 ml physiological saline, administered at 0.01 ml/BW) was intraperitoneally injected to induce anesthesia. Complete anesthesia was confirmed by tail pinch, and the head was fixed in a stereotaxic apparatus (Narishige, Japan). Following skin incision, 2-3 bur holes corresponding to the hippocampal injection sites were made using an electrical drill against the skull. The cortical surface was exposed and then covered with wet cotton soaked in physiological saline to avoid dryness. All animal experiments described here were approved by the animal care and use committee of the Johns Hopkins University (herpes simplex virus injection) or the University of Miyazaki (lentivirus injection). 

### EEG measurements and calculation of integrated value

To record EEG, a differential amplifier (Cygnus Technology, NC USA) was used; wherein, the positive input was connected to the wire in the pipette, and the negative input was grounded to the ear bar in the stereotaxic frame. Using an amplification rate of 10,000, the low-pass and high-region interception filters were set at 1 Hz and 1 kHz, respectively. The analog-to-digital conversion (PowerLabModel4/20, AD Instruments, Japan) was set to a sampling rate of 1 kHz, and all data obtained from each section were saved on a hard disk. The 4-8 Hz theta band was extracted by real-time calculation as theta oscillation during the injection. The extracted theta band was quantitatively evaluated by integrative analysis. For injection into the dorsal hippocampal CA1 region, two electrode coordinates based on a mouse brain atlas were tested over several trials, and then the cortical insertion positions for all of the experiments were determined as follows: AP: -2.18 mm from bregma; ML: ±1.6 mm from the sagittal suture [21]. In the dorso-ventral direction (DV, depth), the first position of the needle at the cortical surface was determined by EEG detection and was set to zero. As the needle was inserted into the brain, EEG and theta oscillation data were recorded and analyzed at each 0.05-0.1 mm of depth. For regular injection without EEG monitoring, the reported coordinates, AP: -1.94 mm, ML: 1.00 mm, DV: 1.25 mm, were employed in ten injections [22,23]. In addition, we calculated the presumable coordinates, AP: -2.18 mm, ML: ±1.6 mm, DV: 1.00 mm, based on the mouse brain atlas and performed twenty injections without EEG monitoring [24]. Injection sites were analyzed by dye injections in brain sections.

### Dye, latex microsphere, and virus injection

Before insertion, the glass pipette was filled with either pontamine skyblue solution or latex beads (fluorescent carboxylate microsphere 0.10µm, Polysciences Inc., PA USA ) to confirm the needle position or virus solution for gene transfer. After the tip of the glass pipette was positioned at the target location, the injection dye, latex beads, or virus solution was expelled using a microsyringe pump at a rate of 0.2 µl/min for one (dye and latex microsphere) or eight minutes (virus). 

After the injection, the mice were sacrificed immediately (dye) in three days (herpes simplex virus) or more than one week later (lentivirus). For histological analysis, the brain was removed and fixed overnight with a 4% paraformaldehyde-PBS solution followed by a 20% sucrose solution. Fifty-micrometer sequential brain slices were collected using a vibratome (Daiwa light machine REM-700, Japan). The brain slices were mounted onto a glass slide coated with 1% gelatin liquid and 0.1% chromium alum liquid and dried before crystal violet counter-staining. Stained brain slices were observed through a microscope (Nikon Cool scope, Japan). In order to investigate the localization of fluorescent latex microsphere to check injection sites, we use fluorescent microscopy with a rhodamine filter. For standard immunohistochemical methods for GluA1 immuno-staining, brain sections were treated with an anti-GluA1 antibody and observed using a fluorescent microscope (Zeiss Axio Observer, Germany) [18]. 

### Virus preparation

Herpes simplex virus vectors were generated following a previously described method [25,26]. The p1001 (+) vector was used as a shuttle vector. This vector has two promoters, IE4/5 and CMV. We made a construct to express EGFP (Enhanced Green Fluorescent Protein) and rat GluA1 cDNA, which were transcribed by the CMV and IE4/5 promoter, respectively. The p1001(+) shuttle vector was transfected into a host cell line 2-2 using Lipofectamine 2000 (Invitrogen, CA USA); wherein, a helper virus was then infected the next day. After cytopathic effects were observed in the host cells, the cells were collected, and the virus particles were released from the cytoplasm by ultrasonic disruption and freeze/thaw cycles. The virus solutions were repeatedly infected into new host cells to amplify the titer. After collecting infected host cells in the same way, a crude solution containing virus particles was separated by low-speed centrifugation to remove cell debris. The virus particles were then purified by sucrose gradient ultracentrifugation. Subsequently, the particles were concentrated by an additional ultracentrifugation. The virus pellet was suspended with a small amount of PBS(+), made into aliquots, and stored at -80 °C. The titer of the virus solution was evaluated using 2-2 cells.

Lentivirus vectors were also generated following previously published methods [27]. Briefly, a FUGW shuttle vector, which contained EGFP transcribed by the Ubiquitin promoter, delta8.9, and VSVG were triple transfected into HEK293T cells using Lipofectamine 2000 (Invitrogen, CA USA), and the virus-containing medium was collected on the second and third days. After removing rough cell debris using a 0.45μm filter and low-speed centrifugation, the virus solution was concentrated by ultracentrifugation at 154,000 g for 90 minutes. The precipitated virus particles were suspended with a small amount of PBS (+). The vector solutions were divided into aliquots and stored at -80 degree. For LTP rescue experiment in GluA1 knockout mice, EGFP was replaced with ZsGreen for brighter signal to easily identify infected cells, and it was fused with IRES-bicistronic construct expressing mouse GluA1 cDNA. The viruses were titered in HEK293T cells. The virus solution from the same lot was also tested in primary cultured neurons for the titer, toxicity, and expression of the exogenous genes. We usually infected lentivirus constructs into 3-4 weeks old mice brains and investigated 10-14 days later for maximum expression of exogenous proteins. 

### Electrophysiological analysis

Acute hippocampal slices were prepared 1 to 2 weeks after virus injection. The mice were anesthetized with isoflurane, and the brains were rapidly removed and placed into an ice-cold cutting solution (120 mM choline chloride, 3.0 mM KCl, 8.0 mM MgCl_2_, 1.0 mM CaCl_2_, 1.25 mM NaH_2_PO_4_, 28 mM NaHCO_3_, 1.0 mM kynurenic acid, and 25 mM glucose, saturated with 95% O_2_/5% CO_2_). Coronal slices (300 μm thick) were prepared with a vibratome (Leica VT1200, Germany). The slices were recovered in ACSF (125 mM NaCl, 2.5 mM KCl, 1.0 mM MgCl_2_, 2.0 mM CaCl_2_, 1.25 mM NaH_2_PO_4_, 26 mM NaHCO_3,_ and 11 mM glucose, oxygenated with 95% O_2_/5% CO_2_), supplemented with 1.0 mM kynurenic acid at 35 degrees for 1 h, and were then kept in ACSF at room temperature until data collection.

 The slices were placed in a submerged chamber and perfused at room temperature in ACSF supplemented with 100 μM picrotoxin. The CA3 was dissected from each slice immediately before data collection to prevent burst activity. Whole-cell recordings were obtained from the CA1 pyramidal cells under differential interference contrast and fluorescent illumination. An AXOPATCH 200A amplifier and pClamp10 software (Molecular Devices, CA USA) were used for data acquisition. The signals were digitized at 10 kHz and low-pass filtered at 2 kHz, and the liquid junction potentials were left uncompensated. For the voltage-clamp recordings, the intracellular solution contained 130 mM CsMeSO_4_, 0.2 mM EGTA, 20 mM CsCl, 10 mM HEPES, 2.0 mM ATP-Mg, 0.3 mM GTP-Na, 5 mM QX314, and 0.1 mM spermine (pH = 7.2 and osmolality = 285–300 mOsm). Patch pipettes with 7 to 10 MΩ resistance were used for the recordings. Miniature excitatory postsynaptic current (mEPSC) recordings were performed at –70 mV in the presence of 1 μM tetrodotoxin and 50 μM AP-5 in the bath, and the results were analyzed using MiniAnalysis software (Synaptosoft Inc., GA USA). The amplitude threshold for detecting mEPSC events was set at 5 pA. AMPA-mediated EPSCs were obtained by voltage-clamping the cell at -70 mV and electrically stimulating Schaffer collateral inputs (SCs). To induce LTP, the cells were held at 0 mV, while SCs were stimulated at 2 Hz for 200 pulses. Any recordings, in which the access resistance changed by more than 20%, were discarded. All of the experiments and analyses were blindly performed [28].

### Statistics

Data were represented as mean ± SEM. Analyses involving three paired data sets were performed with repeated measures ANOVA. Analyses involving two data sets were performed with a Student’s t test, only if the variances were statistically different. Significance was set as a p-value of less than 0.05. We performed statistical analysis using StatPlus (AnalystSoft, Canada) or R statistical software. 

## Results

### Establishing the electro-injection system

**Figure 2:**
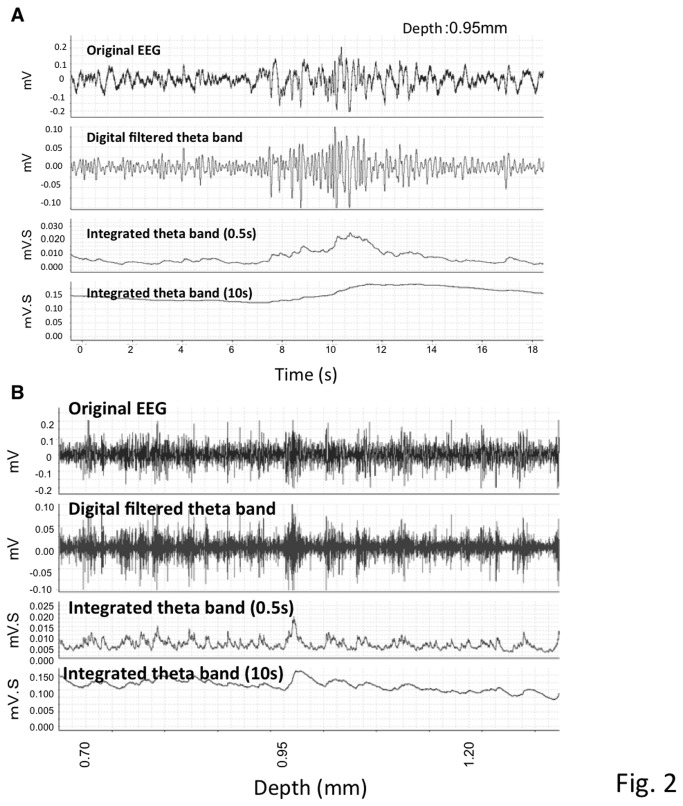
Representative EEG data. **A**. Raw EEG data collected from a recording electrode (top) and filtered EEG after selecting for theta oscillations (4-8 Hz) (middle). An isolated theta oscillation in a 10 sec and 20 sec bin was analyzed using integrated values (0.5 sec and 10 sec durations) at a depth of 0.95mm from the cortex. **B**. Sequential recording of EEG at different depths. The same three types of data are presented as in Figure 2A with changes at different depths indicated at the bottom. Twenty seconds of recording data at each depth were collected, analyzed, and integrated over 0.5 sec and 10 sec.

First, the changes in the extracellular field response when inserting the glass pipette were monitored. The size and frequency of the theta oscillation were compared at each 0.1 - 0.2 mm of depth from the corresponding hippocampal area of the cortical surface. Extracellular field responses were collected for 20 seconds at each depth (Figure 2). Previous reports speculated that, compared to the cortex, the amplitude and frequency of the theta oscillations were relatively high at a particular depth presumed to be the hippocampal CA1 pyramidal cell layer. A second large peak was also observed below the CA1 as previously reported [29,30].

Next, the integrated values of the theta oscillations were calculated in real time at each depth. Monitoring began 0.6 mm below the cortical surface, and the first high value, approximately 0.95 -1.40 mm below the cortex, indicated the hippocampal CA1 pyramidal layer. 

**Figure 3:**
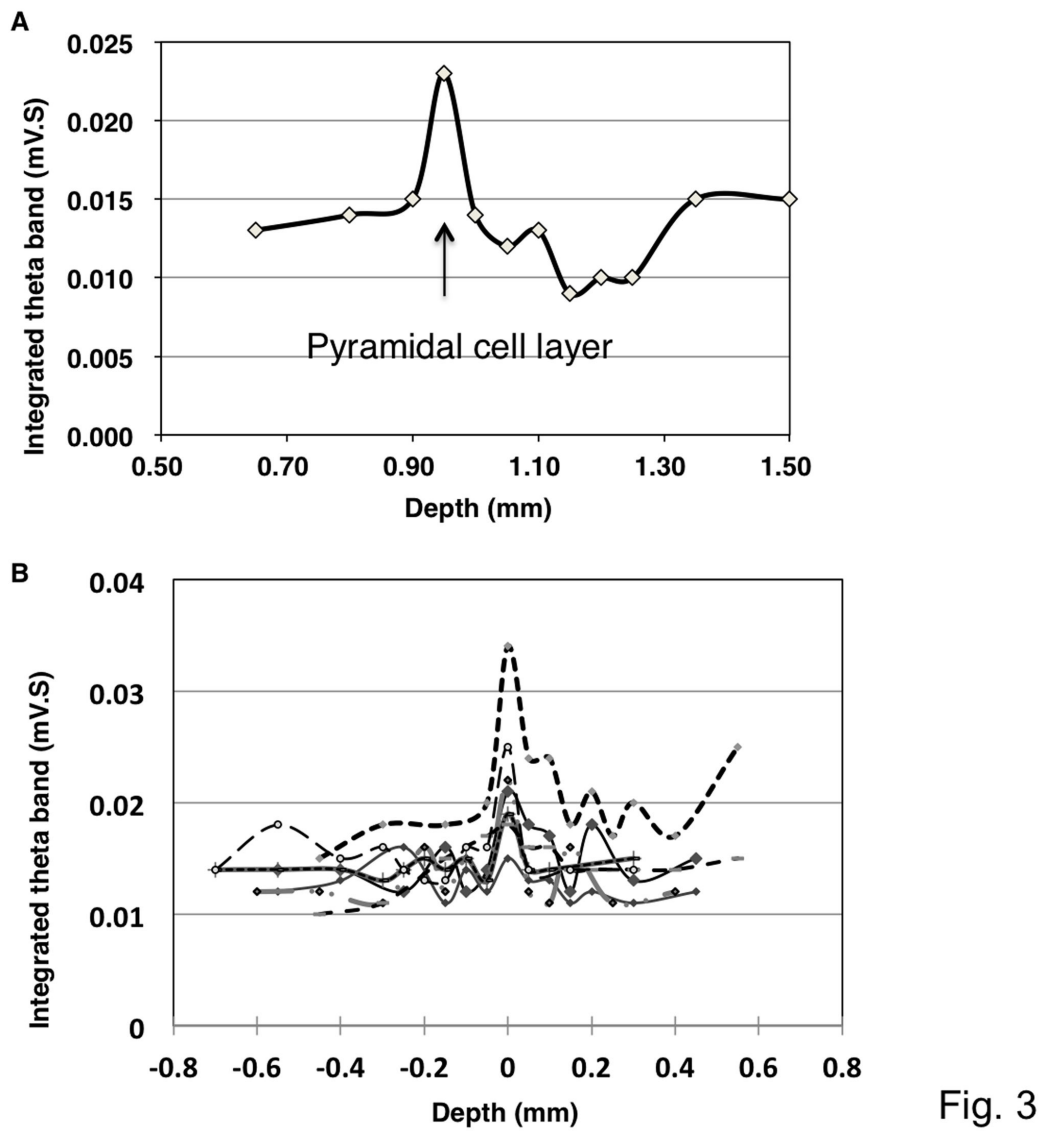
Representative traces showing changes in the integrated value of the theta oscillation at different depths. **A**. A theta oscillation integrated over 0.5 sec indicated the highest value presumably in the pyramidal layer of the hippocampus. After the first peak, the value declined towards the bottom of the stratum radiatum and then increased again. **B**. Superimposition of the integrated values of the theta oscillation. The changes in the integrated values across seven independent experiments were superimposed. All of the data presented a similar pattern of peaks, and these peaks overlapped at a point that was assumed to be the CA1 pyramidal layer.

After monitoring sequential integrative values through the cortex up to the deeper region beneath the CA1 pyramidal layer and determining the presumable location of the CA1 pyramidal layer, the glass electrode was pulled up and re-positioned at the depth of the first large peak in the theta band. Figure 3A demonstrates representative continuous changes in the integrated values at each depth, which shows the highest integrated value at the CA1 pyramidal layer. The later peaks were presumed to be the stratum radiatum and deeper structures in the hippocampus. These patterns were observed in most of our experiments, although the relative size of each peak differed in individual experiments due to different resistances between electrodes, which were determined by the tip diameter and inner solutions (Figure 3B).

### Confirmation of pipette position by injection dye and latex microsphere

**Figure 4:**
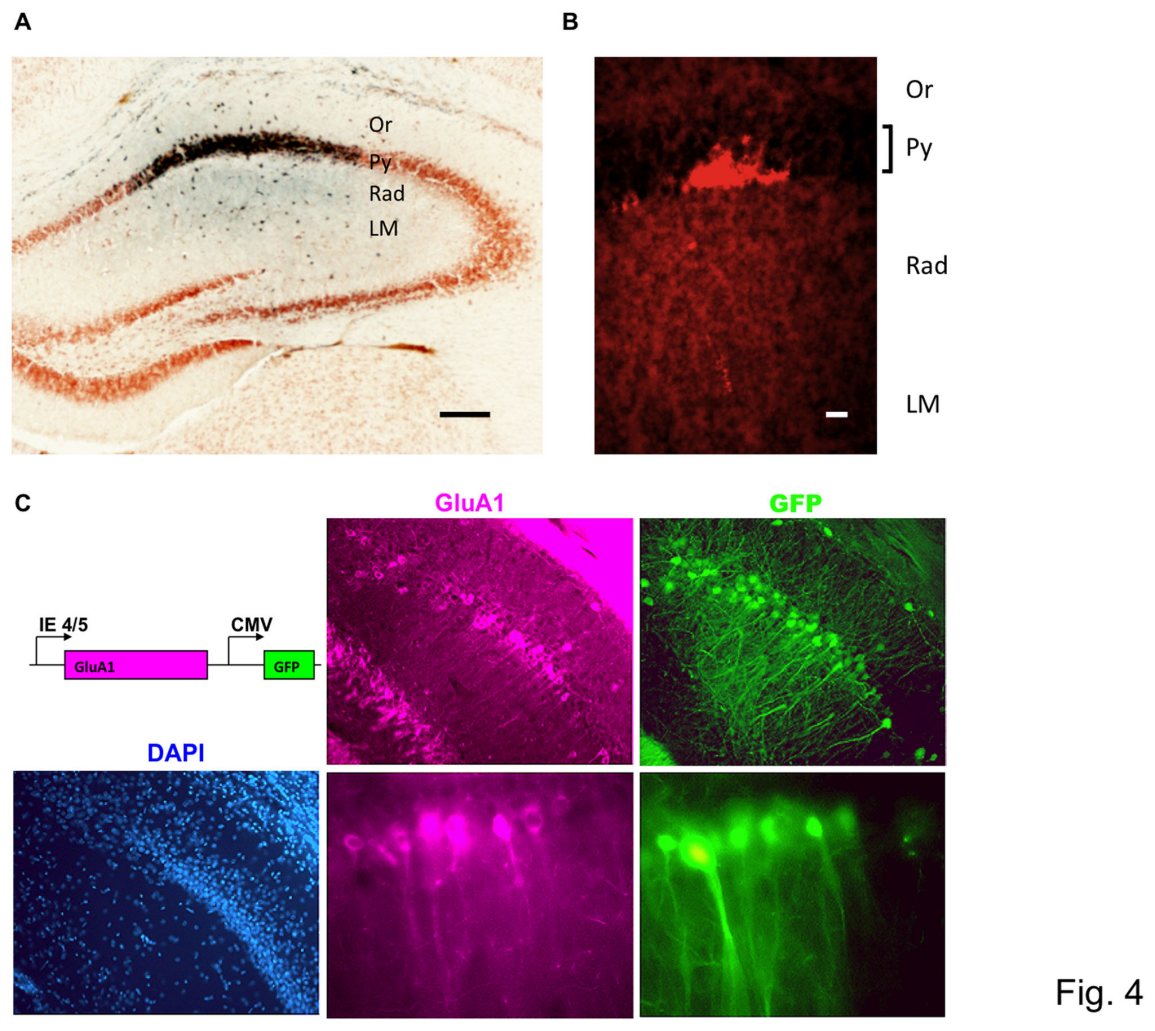
Histological evaluations of injection accuracy and gene expression. **A**. Dye was injected to confirm the position of the glass electrode tip after identification of the CA1 pyramidal layer based on the analysis of the theta oscillation. Pontamine skyblue dye was injected, and the slice was counter-stained with cresyl violet. The dye-injected area was shown as a dark area corresponding to the CA1 pyramidal layer. Scale bar, 200µm. **B**. Latex microspheres demonstrated the precise injection sites in pyramidal cell layer. Injected fluorescent-coated latex microspheres accumulated at the bottom layer in stratum pyramidale. Scale bar, 200µm. Or: stratum orience, Py: stratum pyramidale, Rad: stratum radiatum, LM: stratum lacunosum-moleculare. **C**. Herpes simplex virus infected-neurons expressing EGFP and GluA1 in the hippocampus. A herpes simplex virus vector expressing EGFP and GluA1 simultaneously was injected into the hippocampus of GluA1 knockout mice. The scheme of the expression cassette was indicated in the top left. Three days after the injection, a brain slice from the injected brain was stained with anti-GluA1 antibody (violet). Note that EGFP-positive neurons (green) expressed exogenous GluA1 at various levels in the picture taken in the same focus plane (top: low magnification, scale bar, 100µm; bottom: high magnification, scale bar, 20µm). DAPI: nuclear staining. Scale bar, 100µm.

 The position of the glass electrode tip was confirmed by local injection of pontamine skyblue dye solution at the position of the presumptive CA1 region. Histological analysis of the brain slices demonstrated proper location of the electrode tip in the CA1 layer in most of the experiments (Figure 4A). In addition, the injection of latex microsphere to prevent diffusion more precisely indicated the injection sites in the pyramidal cell layer (Figure 4B).

 We first compared successful (n=22) and failed (n=6, data not shown) injections into the CA1 pyramidal layer and evaluated the efficiency of this approach. Most of the failures resulted from the dye that leaked into the lateral ventricle or from injections that were too deep and reached the dentate gyrus. In the end, the successful injection rate was greater than 75% across all of the experiments (n=28). In the last 14 experiments after improving the system, the successful rate has reached up to 92.8% (success: failure = 13:1). Injection into the CA1 by the same investigator without EEG monitoring was also conducted to evaluate the efficiency of our system. Ten injections following the previous literatures and twenty injections based on the mouse brain atlas were performed [22-24]. The rates of successful injection without EEG monitoring were 40% (n=10, success: failure = 4:6) and 35% (n=20, success: failure = 7:13) following the previous literatures and mouse brain atlas, respectively.

**Figure 5:**
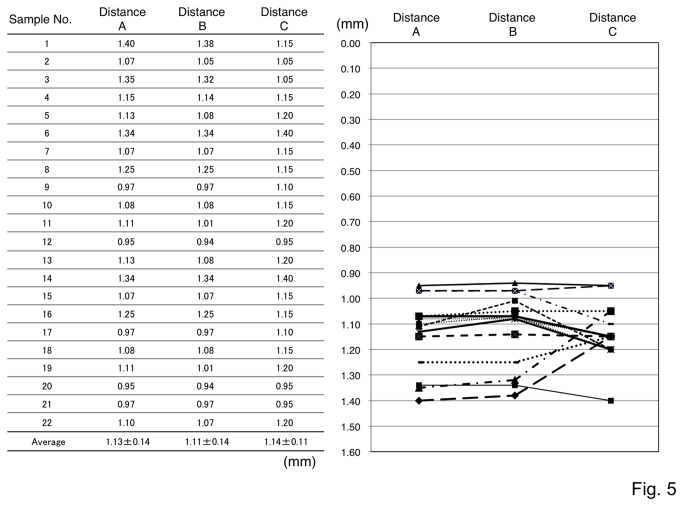
Comparison of distance from the cortical surface to the CA1 pyramidal layer in histological analyses and practical injections.

The distance from the cortical surface to the CA1 pyramidal layer in the brain slices from each injection was then measured. If the cortical surface was damaged by the inserted electrode or the drilled bur holes, the measurement was taken from the presumptive surface estimated by the surrounding cortical surfaces as compensative　distances (Distance A) compared to actual distances without compensation (Distance B) (Figure 5). 

In 22 successful injections confirmed by dye injection, the distances (mm) from the cortical surface to the CA1 pyramidal layer were measured in histological brain sections. If the cortical surfaces were damaged while making the bur holes or inserting the electrodes, the presumable cortical surface lines were set by the neighboring cortex (Distance A). Distance B indicated the actual distances measured from the insertion points to the CA1 pyramidal layers without compensatory correction of cortical surface. Each insertion depth measured by the stereotaxic apparatus was indicated as Distance C. Three different measurements from each injection were shown in a graph on the right side. There were no significant differences between distances in three groups (repeated measures ANOVA, *P>0.05*).

 There were no significant differences in the distances from the histological cortical surface to the CA1 pyramidal layer between successful (1.13 ± 0.14 mm) and failed (1.19 ± 0.16 mm) injections (*P*>0.05). Even in successful injections, however, there were some variations in the distance (0.95 - 1.40mm) from the cortical surface to the CA1 pyramidal layer in the mice used in this study, despite the fact that we used similarly aged inbred mice. These variations may be due to variation in sizes of the brain, brain swelling, or decompression from the pre-treatment with mannitol during the surgery. 

 The depth of the inserted electrode, which was measured by the stereotaxic apparatus, was also similar in both groups (Distance C, successful injections: 1.14 ± 0.11 mm, failed injections: 1.13 ± 0.18 mm, *P*>0.05). In the successful injection group, however, gaps up to 0.30 mm were noted between the histological distances and practical measurements of the inserted electrode, in spite of the absence of significant differences (Figure 5). The results indicate that practical injections have potential complications if simple formula coordinates are used. In our measurement in histological samples, the brain may be slightly shrunk and shortened in Distances A and B; however there were no significant statistical differences between all three groups including A/B and C (repeated measures ANOVA, *P*>0.05).

### Gene transfer into CA1 pyramidal cells

**Figure 6:**
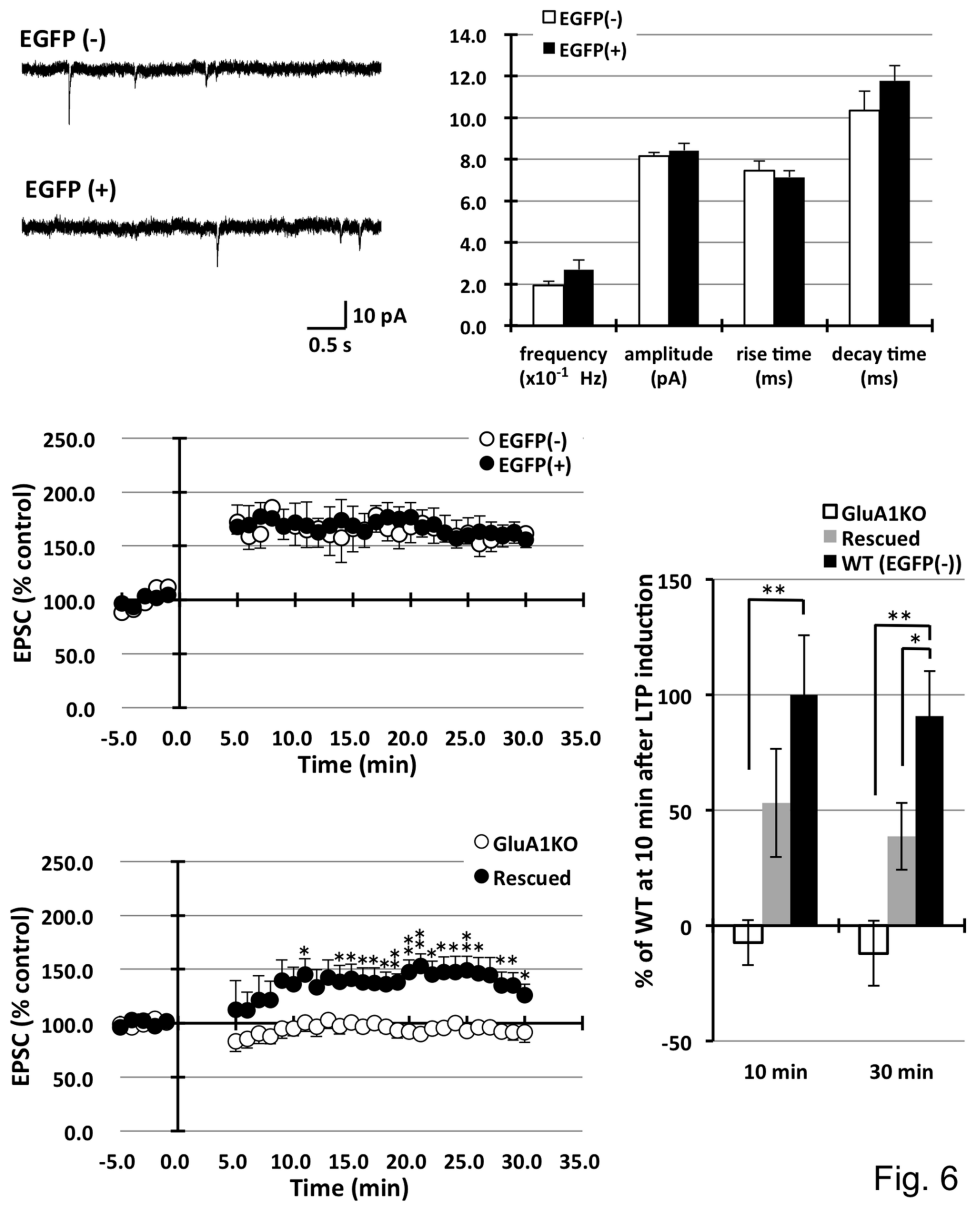
Electrophysiological analysis of lentivirus-infected neurons. EGFP-expressing lentivirus vectors were stereotaxically injected into the CA1 pyramidal neurons, and patch clamp analyses were carried out on infected and non-infected neurons. **A**. **B**. Analysis of the mEPSCs showed comparable frequency, amplitude, and decay time compared to the non-infected neurons. Non-infected cells: EGFP(-) n=7, infected cells: EGFP(+) n=8, **C**. Pairing-induced LTP was not significantly different between the two types of neurons. EGFP(-) n=6, EGFP(+) n=7. **D**. The injection of ZsGreen lentivirus vector expressing GluA1 into hippocampus CA1 had partially rescued LTP in GluA1 knockout mice. (*P<0.*05*, **P<0.01) **E**. Statistical analysis of LTP in wild (EGFP (-)), rescued, and GluA1 knockout mice in 10 min and 30 min after LTP induction. LTP was partially rescued by GluA1-expressing lentivirus infection in GluA1 knockout mouse. (*P<0.*05*, **P<0.01).

 The method was applied to inject EGFP-expressing lentiviruses. Because these viruses preferentially transduce neuronal cells, infected pyramidal neurons in the CA1 specifically expressed the EGFP signal. EGFP expression spread into the dendrites, axons, and entire cell body. After waiting for more than one week for recovery from surgery, neuronal function was tested using electrophysiological techniques. Following the preparation of acute brain slices, clear EGFP-expressing pyramidal neurons were discovered under fluorescent microscopy. Infected neurons were distributed approximately 1 mm around the injection sites. The mEPSCs between infected and non-infected neurons were compared. Patch clamp analysis revealed that lentivirus-infected EGFP-expressing neurons exhibited similar mEPSC frequencies (non-infected 0.19 ± 0.22, infected 0.27 ± 0.05 ×10^-1^Hz), amplitudes (8.15 ± 0.18 vs. 8.42 ± 0.34 pA), rise times (7.46 ± 0.47 vs. 7.14 ± 0.31 ms), and decay times (10.34 ± 0.93 vs. 11.76 ± 0.74 ms) compared to the AMPA-type glutamate channel properties in non-infected neurons, as shown in Figures 6A and B. Furthermore, LTP was induced in infected neurons using a paired induction protocol and similar increases in EPSCs were observed (EPSC amplitude at 30 min after stimulation: non-infected 161.7 ± 13.3, infected 155.9 ± 10.8 % control) (Figure 6C). Lentivirus-infected cells functioned similarly to non-infected neurons with minimal toxic effects. In the lentivirus system, toxicity was occasionally observed during the virus titration experiments in infected primary culture neurons. This toxicity is presumably caused by low purity of the virus, i.e., the carryover of cell debris and/or serum in the culture media. Therefore, newly prepared lot of virus was used for each injection.

It is known that AMPA-type glutamate receptors are major excitatory neurotransmitter receptors in the central nervous system, and GluA1 subunit of these receptors plays a crucial role to express LTP in CA1 hippocampus. Therefore, the rescue of LTP expression in GluA1 knockout mice CA1 hippocampus was attempted, in which LTP expression was completely abolished [31,32]. IRES-containing bicistronic lentiviruses expressing GluA1 vectors were injected into the CA1 hippocampus. Infected neurons that simultaneously expressed EGFP and mouse GluA1 protein were easily identified for the following patch clamp analysis. LTP from infected neurons were measured and approximately half size of rescued LTP was successfully obtained (10min; wild type LTP = 100.0±25.5, rescued LTP = 53.2±23.5, GluA1 knockout LTP = -7.4±9.8, 30min; wild type LTP = 90.8±19.5, rescued LTP = 38.6±14.5, GluA1 knockout LTP = -12.1±14.1). 

Our system was also employed to introduce additional exogenous genes into the hippocampal CA1 pyramidal neurons. The herpes simplex gene transfer systems were chosen because these viruses are neuronal-oriented virus vector systems with relatively lower toxicity compared to other viral gene transfer systems, such as Sindbis and Adenoviruses [33]. 

Using the herpes simplex virus vector system, a shuttle vector was designed to simultaneously express EGFP and GluA1 subunit of the AMPA-type glutamate receptor. GluA1 knockout mice were used in this experiment to distinguish from endogenous GluA1 proteins. Gene expression was evaluated using immunostaining analysis on the third day for maximal expression following the injection. Because two promoter systems were used to express EGFP and exogenous GluA1 proteins individually, simultaneous expression of the two genes in the same infected neurons was evaluated. Most EGFP-expressing neurons also expressed GluA1, although the level of each expression varied in the same cells. It was clearly demonstrated by pictures in the same focus plane (Figure 4C). Toxicity in the infected neurons was observed 5 days after infection as previously reported [33].

## Discussion

Monitoring the theta oscillation revealed a peak amplitude in the hippocampal CA1 layer, a finding consistent with a previous report [29]. To obtain objective results in each experiment, the integrated values of the spontaneous theta oscillations, measured every 0.05 or 0.1 mm from the brain surface to the stratum moleculare and the stratum radiatum in CA1, were calculated. The integrated values of the theta oscillations showed a maximum near the pyramidal layer and a minimum near the stratum radiatum, and the values tended to increase thereafter. The size of the theta oscillations in the individual experiments varied; however, the relative integration value enabled us to minimize the differences between experiments and animals. In this study, the dorsal hippocampus was targeted, where the distance from the cortical surface to the CA1 pyramidal region was thought to be ~1.1 mm according to the mouse brain atlas [21]. This distance was similar to the average measurement in our histological analysis. However, there was a 0.45mm variation, with a range of 0.95 - 1.40 mm among animals, which could impede accurate injections using formula coordinates. The cortical surface was set as zero, where the first EEG responses were detected. The practical distances of the injection sites measured by the stereotaxic apparatus differed by up to 0.30 mm compared to the histological distance. In 7 out of the 22 successful injection cases, the gap between the histological distance and the practical measured distance was greater than 0.1 mm, which could be critical for an accurate injection into the thin CA1 pyramidal layer. 

These differences may be caused by a misalignment of the zero point at the cortical surface due to brain swelling/decompression or damage to the cortical surface during surgery. In this study, the focus was injections into the dorsal hippocampus. In the case of multiple injections into different hippocampal regions, including the ventral hippocampus, the lateral and dorsoventral coordinates will vary. In particular, the depth of the dorsoventral directions distinctly changes with each position in the CA1 layer of dorsal hippocampus by 1.5.- 3.75 mm [34]. Our reported method may be flexibly adjusted for multiple injections with different depths into widespread hippocampal regions. In addition, our method reveals two times more efficiency compared to the regular injection without EEG monitoring with regard to accuracy.

In this study, the appropriate virus vector systems for neuroscience research were also examined. Lentivirus and herpes simplex vector systems were employed due to their preference for infecting neurons. The packaging capacity of each virus is dependent upon the size of the original viruses, e.g., lentivirus is capable of expressing up to an 8-kb exogenous gene [35]. In addition, lentiviral vectors are generated by reverse transcriptase, and the viral genome transcript integrates into the host genomic DNA. Therefore, it is difficult to generate high-titer virus vectors with a large cDNA insert, as would be required for glutamate receptors (>3 kb). In comparison, the herpes simplex virus has an initially large 40 kb capacity and can grow episomally without integration into the genomic DNA. Hence, it can be generated with a high titer and can stably express a large exogenous gene [33,35].

The preparation of lentivirus vectors can be completed in 4-5 days. This simple procedure is a prominent advantage compared to the herpes simplex virus vectors, where it is necessary to repeat the infection of the host cells several times to increase the virus titer. Several purification steps are also required because the viruses accumulate in the cytoplasm, and the entire process can take approximately two weeks. 

The lack of toxicity with lentivirus is characteristic, as it is used to generate transgenic mice via the integration of inserts into genomic DNA [27]. Other virus vectors are toxic at various levels and intervals, i.e., herpes simplex virus induces cell death in one week, although EGFP signals can be visualized on the following day after infection. In contrast, lentivirus requires about one week for maximum gene expression. Thus, according to the characteristic features of each virus system, it is important to select the most suitable viral vector for each experiment.

 In this study, we have chosen the herpes simplex virus system to express an AMPA-type glutamate receptor in the CA1 pyramidal neurons. Although the virus has two promoters to express EGFP and GluA1 separately, most infected neurons express both EGFP and GluA1 at various levels. Moreover, by infecting one relatively small area, such as the amygdala, the bilateral injection of viral vectors would allow for behavioral studies to be conducted in mice [36]. Our system will be useful not only for injecting the regions which prominently express theta oscillation, such as the hippocampus CA1 and amygdala, but also for nuclei which express a characteristic firing pattern, such as the subthalamic nucleus [37]. 

The lentivirus-infected neurons were also functionally characterized using electrophysiology. Compared to non-infected neurons, the infected CA1 hippocampal pyramidal neurons showed similar electrophysiological properties, such as mEPSCs and LTP induction, with no signs of toxicity. In addition, the hippocampal LTP was partially rescued in GluA1 knockout mice, in which LTP was completely abolished. Rescued LTP was approximately half of the normal level probably due to a relatively lower expression level of expressed GluA1 protein compared to endogenous proteins. Since lentivirus is a retrovirus and integrates genomic DNA medicated by reverse transcriptase, longer inserts, such as ZsGreen and GluA1, cause low packaging efficiency and low titer in virus vectors generation [38]. The effort for increasing the titer of lentivirus vectors could restore size of LTP as wild type level.

By observing the function of a complicated neuronal network *in vivo*, our study demonstrates that a gene transfer technique using viral vectors and an accurate injection method to target neurons will be useful for a wide range of applications in the field of neuroscience.
